# Wall shear stress and oscillatory shear index measured using ultrasound vector flow imaging in the femoropopliteal artery of adults without peripheral artery disease

**DOI:** 10.3389/fsurg.2025.1597407

**Published:** 2025-10-27

**Authors:** Meiying Gao, Haining Zheng, Rui Zhao, Yan Liu, Lizhu Fang, Yigang Du, Chaoyang Wen, Yisha Tong

**Affiliations:** 1 Department of Ultrasound, Peking University International Hospital, Beijing, China; 2 Department of Ultrasound, Guang’anmen Hospital, China Academy of Chinese Medical Sciences, Beijing, China; 3 Ultrasound R&D Department, Shenzhen Mindray Bio-Medical Electronics Co., Ltd., Shenzhen, China; 4 Department of Vascular Surgery, Austin Hospital, University of Melbourne, Melbourne, VIC, Australia

**Keywords:** atherosclerosis, femoropopliteal artery, occlusion, oscillatory shear index, plaque, relative residence time, ultrasound, vector flow imaging

## Abstract

**Objective:**

To measure wall shear stress (WSS), oscillatory shear index (OSI) and relative residence time (RRT) at multiple locations along the femoropopliteal artery in adults without peripheral artery disease (PAD), and to investigate whether low WSS, high OSI and/or high RRT are present in regions with a high prevalence of atherosclerotic lesions.

**Materials and methods:**

A total of 116 presumed healthy adult volunteers were recruited. Ankle-brachial index and lower-limb arterial duplex ultrasound assessments were performed to exclude participants with PAD. Maximum WSS, mean WSS, and OSI were measured at the near and far walls of ten arterial sites along the femoropopliteal artery using ultrasound vector flow imaging (VFI). RRT was calculated from OSI and mean WSS values obtained via VFI.

**Results:**

A total of 113 participants (46 males and 67 females; 222 lower limbs), with a mean age of 37.6 years, were included in the analysis. Compared to the overall mean values across all 20 measurement sites, significantly higher OSI and higher RRT were recorded at the posterior wall of the common femoral artery (*P* < 0.01), while significantly lower WSS, higher OSI, and higher RRT were observed at the posterior wall of the femoral artery bifurcation (*P* < 0.01). The distal superficial femoral artery (SFA) at the adductor canal demonstrated significantly lower WSS and higher OSI at both near and far walls (*P* < 0.01). When comparing hemodynamic indices between sexes, males exhibited significantly lower WSS at several near-wall sites, significantly higher OSI at multiple near- and far-wall sites, and significantly higher RRT at the near wall of the proximal SFA and the far wall of the profunda femoris artery (*P* < 0.05).

**Conclusions:**

Variations in WSS, OSI, and RRT are evident along the femoropopliteal artery in adults without PAD. Notably, the distal SFA at the adductor canal demonstrated significantly lower and more oscillatory WSS, consistent with the region's reported high prevalence of atherosclerotic plaque and occlusion. Whether these hemodynamic patterns contribute to lesion development later in life remains uncertain and warrants further investigation.

## Introduction

Atherosclerosis is a systemic arterial disease affecting multiple vascular territories, including the coronary, carotid, renal, and lower limb arteries, and is the leading cause of mortality and morbidity worldwide (1–3). The development of atherosclerotic plaques is a complex, multifactorial process influenced by systemic factors, local hemodynamic forces, and the arterial wall's biological response to these factors (4–8).

Wall shear stress (WSS) refers to the tangential force per unit area exerted by blood flow on the endothelial surface of the vessel wall and arises from friction between blood flow and the arterial wall (Equation 1), reflecting both the intensity and direction of blood flow near the wall. WSS is the primary hemodynamic factor influencing the development and progression of atherosclerosis. Low and oscillatory WSS are strongly associated with atherosclerotic plaque formation (9–17).

$$\mathrm{WSS}=\mu {\left(\frac{\partial u}{\partial n}\right)}_{\mathrm{wall}}$$ (1)

Where: *μ* is the dynamic viscosity of blood, and $\frac{\partial u}{\partial n}$ is the velocity gradient perpendicular to the wall. The subscript “wall” denotes that the shear stress is measured on the vessel wall.

Oscillatory shear index (OSI) is a dimensionless parameter quantifying the variation in WSS vector direction during a cardiac cycle (Equation 2), ranging from 0 (purely unidirectional flow) to 0.5 (purely oscillatory flow). A high OSI indicates greater directional oscillation. Elevated OSI values are linked to disturbed, oscillatory flow, with localized zones often corresponding to sites prone to atherosclerosis (18, 19).

$$\mathrm{OSI}=\frac{1}{2}\left(1-\frac{\left|\int_{0}^{T}{\vec{\tau }}_{w}(t)dt\right|}{\int_{0}^{T}|{\vec{\tau }}_{w}(t)|dt}\right)$$ (2)

Where: ${\vec{\tau }}_{w}(t)$ is instantaneous wall shear stress vector at time *t*, and *T* is the time duration of the cardiac cycle.

Relative residence time (RRT) is derived by combining WSS and OSI, reflecting the duration blood particles remain near the vessel wall (Equation 3). RRT increases with higher OSI and lower time-averaged WSS, indicating that disturbed, slow, and oscillatory flow prolongs residence time. Strong spatial correlations have been reported between RRT and atherosclerotic lesion prevalence (20–22).

$$\mathrm{RRT}=\frac{1}{(1-2\mathrm{OSI})\cdot \mathrm{WS}S_{\mathrm{mean}}}$$ (3)

Where: OSI is the oscillatory shear index, and $\mathrm{WS}S_{\mathrm{mean}}$ is the time-averaged mean wall shear stress.

Using reconstructed vascular geometries, WSS and OSI can be calculated mathematically with computational fluid dynamics (CFD). This approach can be combined with imaging modalities such as magnetic resonance imaging (MRI) (23), computed tomography (CT) (24), CT angiography (25), and intravascular ultrasound elastography (26). More recently, high-frame-rate ultrasound vector flow imaging (VFI) has been developed to evaluate flow characteristics, including WSS and OSI measurement (27–29). A strong correlation has been demonstrated between velocity vectors obtained by VFI and those calculated by CFD (30). Compared with MRI, CT, and intravascular elastography, ultrasound VFI is non-invasive, cost-effective, and free of risks associated with ionizing radiation or contrast agents. It can also be safely repeated when clinically indicated.

This study aimed to measure WSS, OSI, and RRT at multiple locations along the femoropopliteal artery in adults without peripheral artery disease (PAD) and to examine whether low WSS, high OSI, and/or high RRT occur at sites with a known high prevalence of atherosclerotic lesions.

## Methods

The study was approved by the Peking University International Hospital Medical Ethics Committee (2022-KY-0053-01). One hundred sixteen presumed healthy adult volunteers were recruited for the study between May 2022 and March 2023. Informed consent was obtained from all participants.

All 116 volunteers completed a questionnaire covering demographic information, risk factors for atherosclerosis, and medical history, including smoking, hypertension, hyperlipidaemia, diabetes, cardiovascular disease, cerebrovascular disease, and chronic renal disease. Ankle-brachial index (ABI) measurement and lower limb arterial duplex ultrasound scans were performed to exclude participants with PAD. VFI images of the femoropopliteal artery were captured, and maximum WSS, mean WSS, and OSI were subsequently measured. RRT was calculated from OSI and mean WSS values obtained via VFI.

### ABI measurement and arterial duplex ultrasound scan of the femoropopliteal artery

A Spead Angiolab 2 Phlebolab Vascular Diagnostic System (Atys Medical, Soucieu en Jarrest, France) was used to conduct the ABI test. The index was calculated by dividing the higher ankle systolic blood pressure (between dorsalis pedis artery pressure and posterior tibial artery pressure) by the higher brachial systolic pressure on each side.

A Mindray Resona 7 ultrasound system (Shenzhen Mindray Bio-Medical Electronics Co, Ltd, Shenzhen, China) with a 3–9 MHz linear array transducer (L9-3U) was used for the arterial ultrasound examination of the femoropopliteal artery. The arterial duplex ultrasound scan was performed to exclude cases with intima-media thickness (IMT) >1 mm, minor plaque or arterial stenosis, based on the Cossman ultrasound criteria (31).

### WSS and OSI measurement of the femoropopliteal artery

VFI images of the femoropopliteal artery were obtained with the Resona 7 ultrasound system. Since the Resona 7 ultrasound system was equipped with an older version of software that can measure WSS, but not OSI, WSS and OSI were subsequently measured using a Mindray Resona R9T ultrasound system (Shenzhen Mindray Bio-Medical Electronics Co, Ltd, Shenzhen, China). This system, with upgraded software, was capable of measuring maximum WSS, mean WSS, and OSI from VFI images transferred from the Resona 7.

#### VFI image acquisition

**Figure 1:**
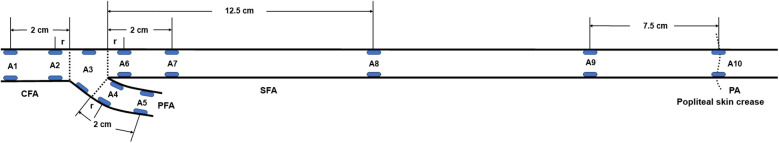
Locations of the femoropopliteal artery for maximum WSS, mean WSS, and OSI measurements (blue ellipses). A1: Common femoral artery (CFA), 2 cm proximal to the femoral artery bifurcation; A2: CFA, one radius proximal to the bifurcation; A3: At the bifurcation; A4: Profunda femoris artery (PFA), one radius distal to the bifurcation; A5: PFA, 2 cm distal to the bifurcation; A6: Superficial femoral artery (SFA), one radius distal to the bifurcation; A7: SFA, 2 cm distal to the bifurcation; A8: SFA, 12.5 cm distal to the bifurcation; A9: SFA, 7.5 cm proximal to the popliteal skin crease; A10: Popliteal artery (PA), at the popliteal skin crease. WSS, wall shear stress; OSI, oscillatory shear index, r: radius.

Grayscale ultrasound was first used to display the longitudinal view of the targeted artery. The “V Flow” tab was activated to enter the VFI mode, and a 1.5-s VFI cine loop was recorded. Acquired VFI cine loops covered A1–A10 of the femoropopliteal artery (Figure 1). A linear array ultrasound transducer with a length of 5 cm was used to estimate the mid and distal superficial femoral artery (SFA) sites (A8 and A9). A8 was located 12.5 cm distal to the SFA origin (≈2.5 transducer lengths), while A9 was located 7.5 cm proximal to the popliteal skin crease (≈1.5 transducer lengths). All VFI images were captured by MG, HZ, RZ, and LF.

**Figure 2:**
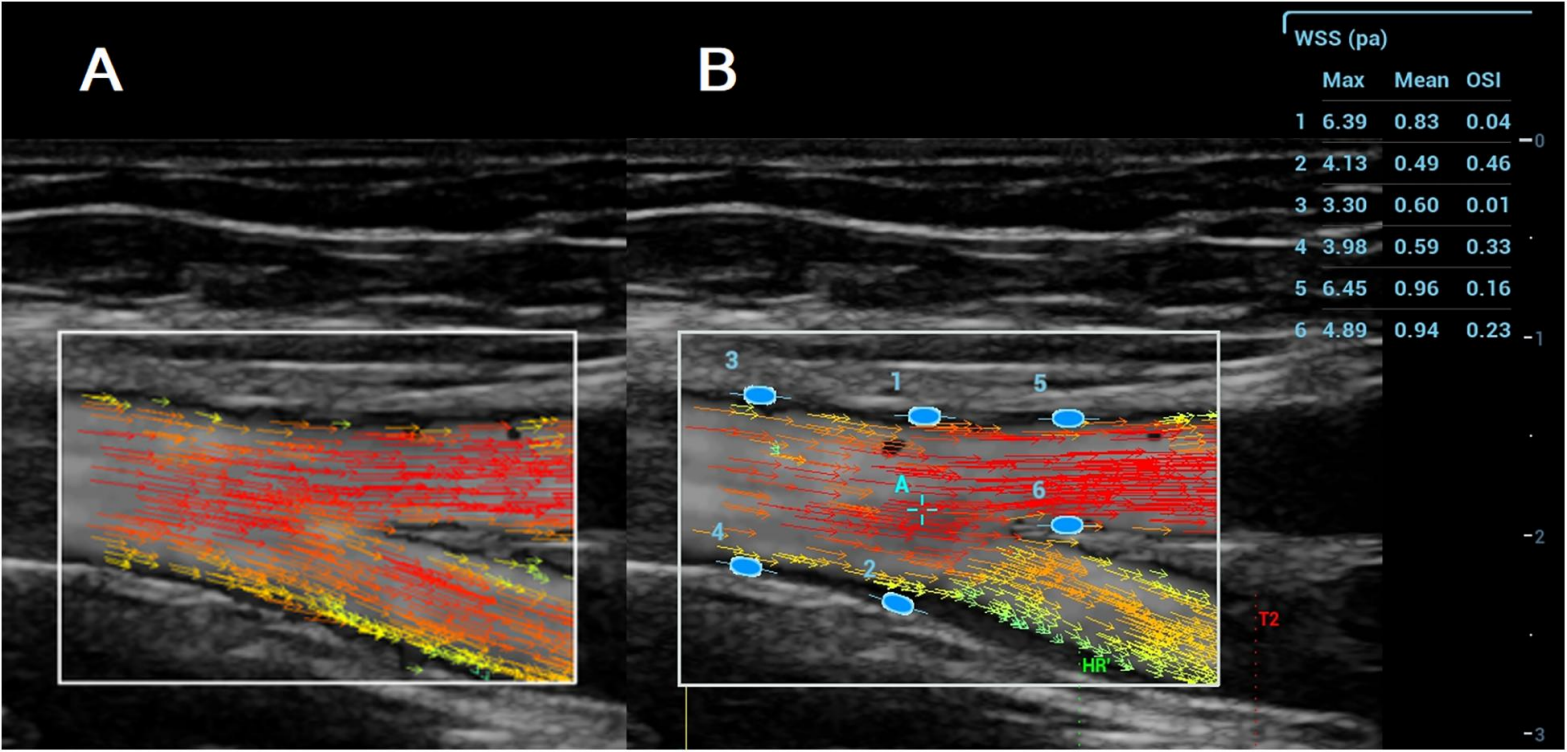
**A** (left): VFI image of the femoral artery bifurcation recorded with the resona 7. Arrows in different colours represent blood flow direction and velocity; **B** (Right): Maximum WSS, mean WSS, and OSI measured with the Resona R9T. Numbers 1–6 indicate locations for maximum WSS, mean WSS, and OSI measurement, along with their corresponding results for maximum WSS, mean WSS, and OSI.

In the VFI image, a series of arrows in different colours represented blood flow direction and velocity (Figure 2A). VFI cine loops were saved on the ultrasound system and transferred to a USB drive.

#### WSS and OSI measurements

VFI cine loop files recorded on the Resona 7 were transferred via USB and analyzed on the Resona R9T system. After selecting a target frame, the operator tapped [WSS], which generated a green ellipse on the screen. The ellipse was positioned and oriented at the vessel wall (A1–A10), and pressing [Set] fixed the measurement site. Maximum WSS, mean WSS, and OSI were displayed in a results window on the right side of the screen (Figure 2B). Measurements were obtained at both the near and far walls of each site. All measurements were performed by MG and YL.

#### RRT calculation

RRT was calculated from OSI and mean WSS values according to (Equation 3) in the Introduction. OSI and $\mathrm{WS}S_{\mathrm{mean}}$ (mean WSS) were obtained from the ultrasound system. RRT approaches infinity when OSI equals 0.5 or when mean WSS $\left(\mathrm{WS}S_{\mathrm{mean}}\right)$ equals 0. OSI and WSS values from the ultrasound system were rounded to two decimal places (Figure 2B). To avoid infinite values in the RRT calculation, OSI was set to 0.4950 when the displayed value was 0.50, and mean WSS was set to 0.0025 when the displayed value was 0.00.

### Statistical analysis

Student's *t*-tests were used to compare the mean values of maximum WSS, mean WSS, OSI and RRT at each of the 20 locations of the femoropopliteal artery with the averaged values of these parameters across all 20 locations. Comparisons of mean maximum WSS, mean WSS, OSI, and RRT values were made between females and males at each of the 20 locations along the femoropopliteal artery. All statistical analyses were conducted using IBM SPSS Statistics V25.0 (Armonk, NY, USA). A *P*-value of < 0.05 was considered statistically significant.

## Results

Of the 116 volunteers, three were excluded due to incomplete data (1 case), diabetes (1 case), and diabetes with cardiovascular disease and chronic renal disease (1 case). An additional four limbs were excluded from the analysis due to IMT >1 mm (2 limbs) and minor plaques (2 limbs).

A total of 113 participants (46 males and 67 females; 222 limbs) with a mean age of 37.6 years (range: 18–57 years) were included in the final analysis. Participant characteristics, including ABI results, are summarized in Table 1.

**Table 1:** Characteristics of the 113 participants (222 lower limbs).

Characteristic	Value	
Gender (*N* = 113)		
Male	46	40.7%
Female	67	59.3%
Age (*N* = 113)		
Age	37.6 (10.5)	18–57
Risk factors (*N* = 113)		
Smoking	20	17.7%
Hypertension	8	7.1%
Hyperlipidaemia	4	3.5%
Body mass index (*N* = 113)		
BMI	23.66 (3.48)	15.92–31.25
Ankle brachial index (*N* = 222)		
ABI	1.16 (0.10)	0.90–1.42

Values are presented as *n*, %, or mean (standard deviation), range. BMI, body mass index; ABI, ankle-brachial index; IMT, intima-media thickness.

**Figure 3:**
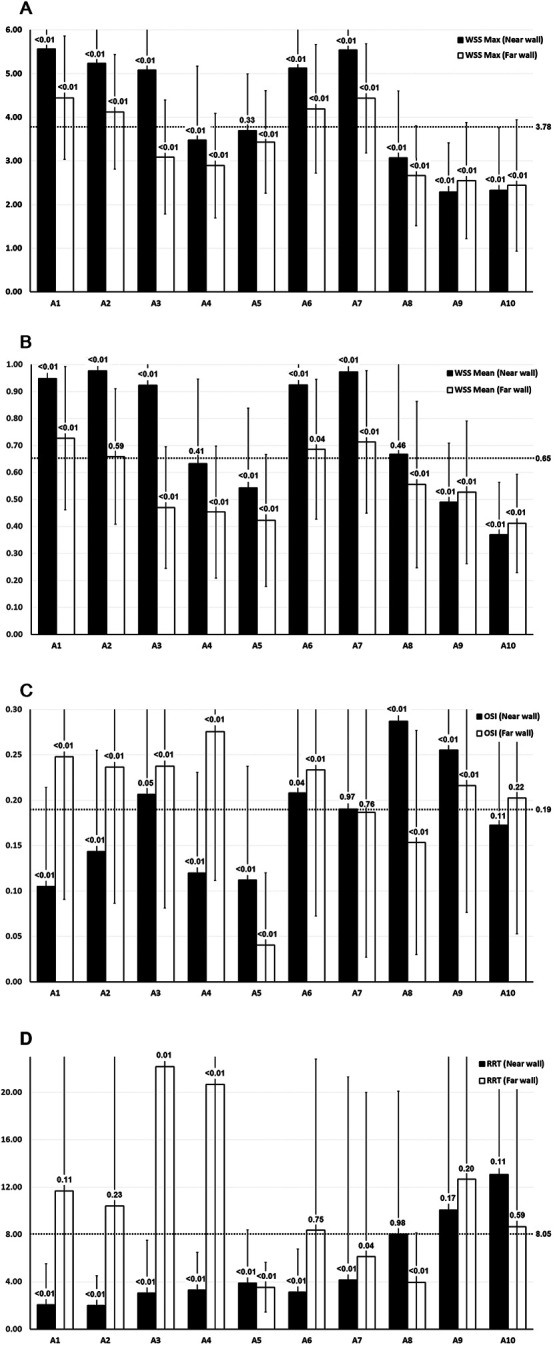
WSS, OSI, and RRT values across the femoropopliteal artery in 222 lower limbs. **(A)** Maximum WSS; **(B)** Mean WSS; C: OSI; D: RRT. A1: CFA, 2 cm proximal to the femoral artery bifurcation; A2: CFA, one radius proximal to the bifurcation; A3: At the bifurcation; A4: PFA, one radius distal to the bifurcation; A5: PFA, 2 cm distal to the bifurcation; A6: SFA, one radius distal to the bifurcation; A7: SFA, 2 cm distal to the bifurcation; A8: SFA, 12.5 cm distal to the bifurcation; A9: SFA, 7.5 cm proximal to the popliteal skin crease; A10: PA, at the popliteal skin crease. WSS: wall shear stress, OSI: oscillatory shear index, RRT: relative residence time, CFA: common femoral artery, PFA, profunda femoris artery; SFA, superficial femoral artery; PA, popliteal artery. Horizontal dotted lines indicate the average values of maximum WSS, mean WSS, and OSI across all 20 locations. Black bars represent measurements at the near wall; white bars represent measurements at the far wall. *P*-values shown above the bars represent the results of *t*-tests comparing values at each location to the overall mean across the femoropopliteal artery.

Maximum WSS, mean WSS, and OSI values were measured at the near and far walls of 10 locations along the femoropopliteal artery in 222 lower limbs. RRT values, calculated from OSI and mean WSS, were also obtained. These results are presented in Table 2 and Figure 3.

**Table 2:** WSS, OSI and RRT values across the femoropopliteal artery in 222 lower limbs.

Locations			CFA		Bifurcation	PFA		SFA				PA	Averaged value
			A1	A2	A3	A4	A5	A6	A7	A8	A9	A10	
Maximum WSS (Pa)	Near wall	Mean	5.56	5.24	5.08	3.48	3.69	5.13	5.54	3.07	2.29	2.33	3.78
		SD	1.54	1.37	1.43	1.69	1.30	1.44	1.43	1.53	1.13	1.43	
		*P*-value	<0.01	<0.01	<0.01	<0.01	0.33	<0.01	<0.01	<0.01	<0.01	<0.01	
	Far wall	Mean	4.45	4.12	3.09	2.89	3.43	4.19	4.44	2.66	2.55	2.44	
		SD	1.41	1.31	1.31	1.20	1.17	1.47	1.25	1.14	1.33	1.50	
		*P*-value	<0.01	<0.01	<0.01	<0.01	<0.01	<0.01	<0.01	<0.01	<0.01	<0.01	
Mean WSS (Pa)	Near wall	Mean	0.95	0.98	0.92	0.63	0.54	0.93	0.97	0.67	0.49	0.37	0.65
		SD	0.32	0.35	0.34	0.31	0.30	0.34	0.33	0.35	0.22	0.19	
		*P*-value	<0.01	<0.01	<0.01	0.41	<0.01	<0.01	<0.01	0.46	<0.01	<0.01	
	Far wall	Mean	0.73	0.66	0.47	0.45	0.42	0.69	0.71	0.56	0.53	0.41	
		SD	0.27	0.25	0.23	0.24	0.24	0.26	0.26	0.31	0.26	0.18	
		*P*-value	<0.01	0.59	<0.01	<0.01	<0.01	0.04	<0.01	<0.01	<0.01	<0.01	
OSI	Near wall	Mean	0.11	0.14	0.21	0.12	0.11	0.21	0.19	0.29	0.26	0.17	0.19
		SD	0.11	0.11	0.13	0.11	0.13	0.13	0.13	0.12	0.13	0.16	
		*P*-value	<0.01	<0.01	0.05	<0.01	<0.01	0.04	0.97	<0.01	<0.01	0.11	
	Far wall	Mean	0.25	0.24	0.24	0.28	0.04	0.23	0.19	0.15	0.22	0.20	
		SD	0.16	0.15	0.16	0.16	0.08	0.16	0.16	0.12	0.14	0.15	
		*P*-value	<0.01	<0.01	<0.01	<0.01	<0.01	<0.01	0.76	<0.01	<0.01	0.22	
RRT	Near wall	Mean	2.06	2.02	3.06	3.32	3.90	3.14	4.16	8.03	10.07	13.06	8.05
		SD	3.46	2.49	4.44	3.17	4.48	3.64	17.14	12.08	21.71	45.96	
		*P*-value	<0.01	<0.01	<0.01	<0.01	<0.01	<0.01	<0.01	0.98	0.17	0.11	
	Far wall	Mean	11.68	10.41	22.18	20.67	3.55	8.36	6.13	3.96	12.67	8.65	
		SD	34.01	29.00	85.62	48.51	2.10	14.44	13.85	4.18	53.35	16.35	
		*P*-value	0.11	0.23	0.01	<0.01	<0.01	0.75	0.04	<0.01	0.20	0.59	

Data are presented as mean, standard deviation (SD). *P*-values were derived from the *t*-test comparing the values at each of the 20 locations with the overall averaged values across all 20 locations. A1: Common femoral artery (CFA), 2 cm proximal to the femoral artery bifurcation; A2: CFA, one radius proximal to the bifurcation; A3: At the bifurcation; A4: Profunda femoris artery (PFA), one radius distal to the bifurcation; A5: PFA, 2 cm distal to the bifurcation; A6: Superficial femoral artery (SFA), one radius distal to the bifurcation; A7: SFA, 2 cm distal to the bifurcation; A8: SFA, 12.5 cm distal to the bifurcation; A9: SFA, 7.5 cm proximal to the popliteal skin crease; A10: Popliteal artery (PA), at the popliteal skin crease. WSS, wall shear stress; OSI, oscillatory shear index; RRT, relative residence time.

*P*-values were derived from *t*-tests comparing the mean values of maximum WSS, mean WSS, OSI, and RRT at each of the 20 locations along the femoropopliteal artery with the overall average values across all locations (3.78 Pa for maximum WSS, 0.65 Pa for mean WSS, 0.19 for OSI, and 8.05 for RRT). These average values are provided in the far-right column of Table 2 and are also depicted as horizontal dotted lines in Figure 3.

**Figure 4:**
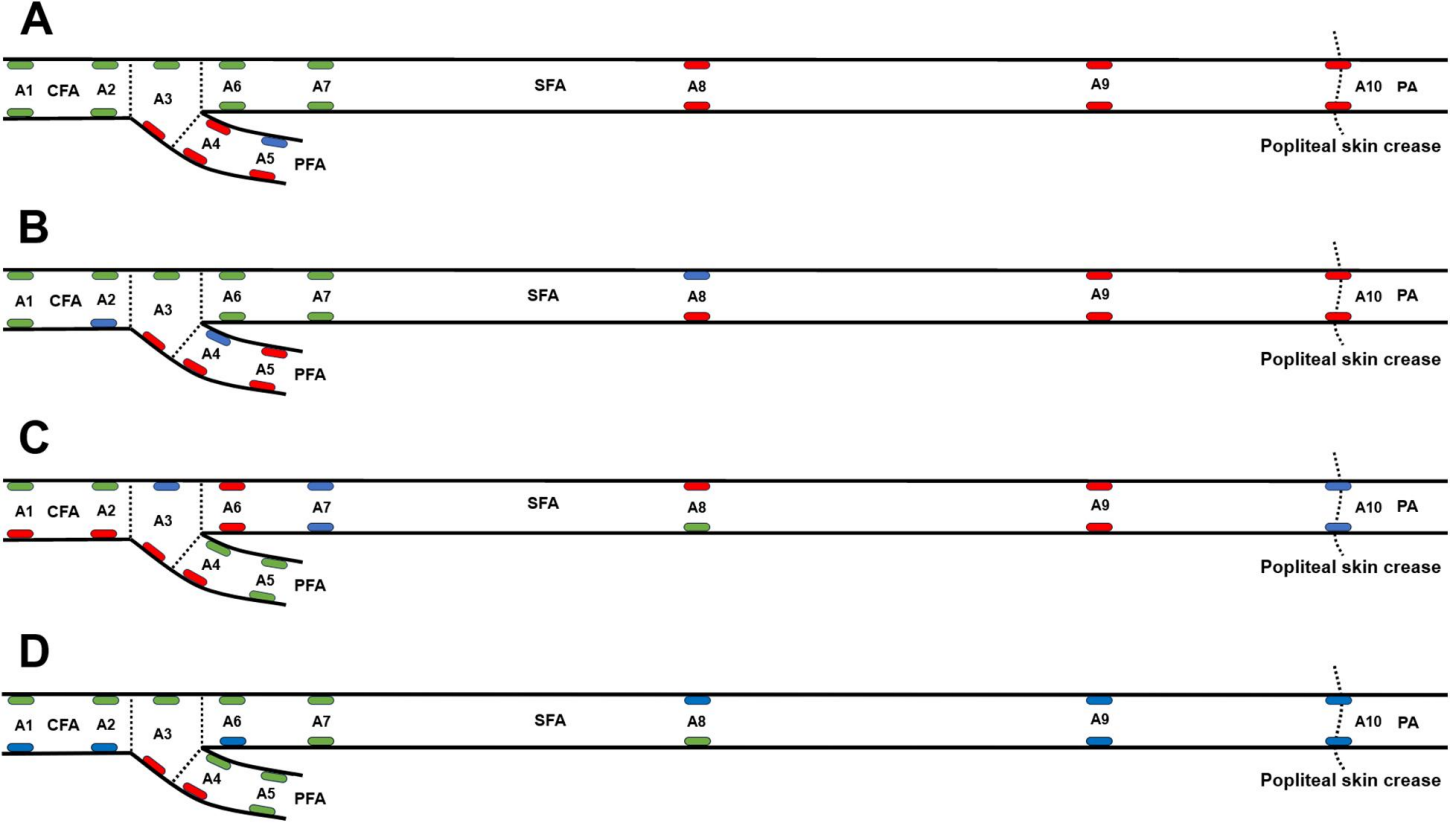
WSS, OSI, and RRT at 20 locations along the femoropopliteal artery in 222 lower limbs, compared with the average values across all locations. **(A)** Maximum wall shear stress (WSS), with green and red ellipses indicating values significantly higher and lower than the average (3.78 Pa), respectively; **(B)** Mean WSS, with green and red ellipses indicating values significantly higher and lower than the average (0.65 Pa), respectively; **(C)** Oscillatory shear index (OSI), with green and red ellipses indicating values significantly lower and higher than the average (0.19), respectively; **(D)** Relative residence time (RRT), with green and red ellipses indicating values significantly lower and higher than the average (8.05), respectively. A1: Common femoral artery (CFA), 2 cm proximal to the femoral artery bifurcation; A2: CFA, one radius proximal to the bifurcation; A3: At the bifurcation; A4: Profunda femoris artery (PFA), one radius distal to the bifurcation; A5: PFA, 2 cm distal to the bifurcation; A6: Superficial femoral artery (SFA), one radius distal to the bifurcation; A7: SFA, 2 cm distal to the bifurcation; A8: SFA, 12.5 cm distal to the bifurcation; A9: SFA, 7.5 cm proximal to the popliteal skin crease; A10: Popliteal artery (PA), at the popliteal skin crease.

Figure 4 presents the results of statistical analysis comparing the values of the maximum WSS, mean WSS, OSI, and RRT at 20 locations of the femoropopliteal artery to the averaged values from all 20 locations. High WSS, low OSI, and low RRT are believed to prevent atherosclerosis and are marked as green, while low WSS, high OSI and high RRT are regarded as promoting atherosclerosis and are marked as red.

In Figure 4A, green ellipses represent maximum WSS values significantly higher than the overall average (3.78 Pa), red ellipses indicate values significantly lower, and blue ellipses indicate no significant difference. In Figure 4B, green ellipses represent mean WSS values significantly higher than the average (0.65 Pa), red ellipses represent significantly lower values, and blue ellipses represent values not significantly different. In Figure 4C, green ellipses indicate OSI values significantly lower than the average (0.19), red ellipses indicate significantly higher values, and blue ellipses indicate no significant difference. In Figure 4D, green ellipses denote RRT values significantly lower than the average (8.05), red ellipses denote significantly higher values, and blue ellipses denote values not significantly different from the mean.

**Figure 5:**
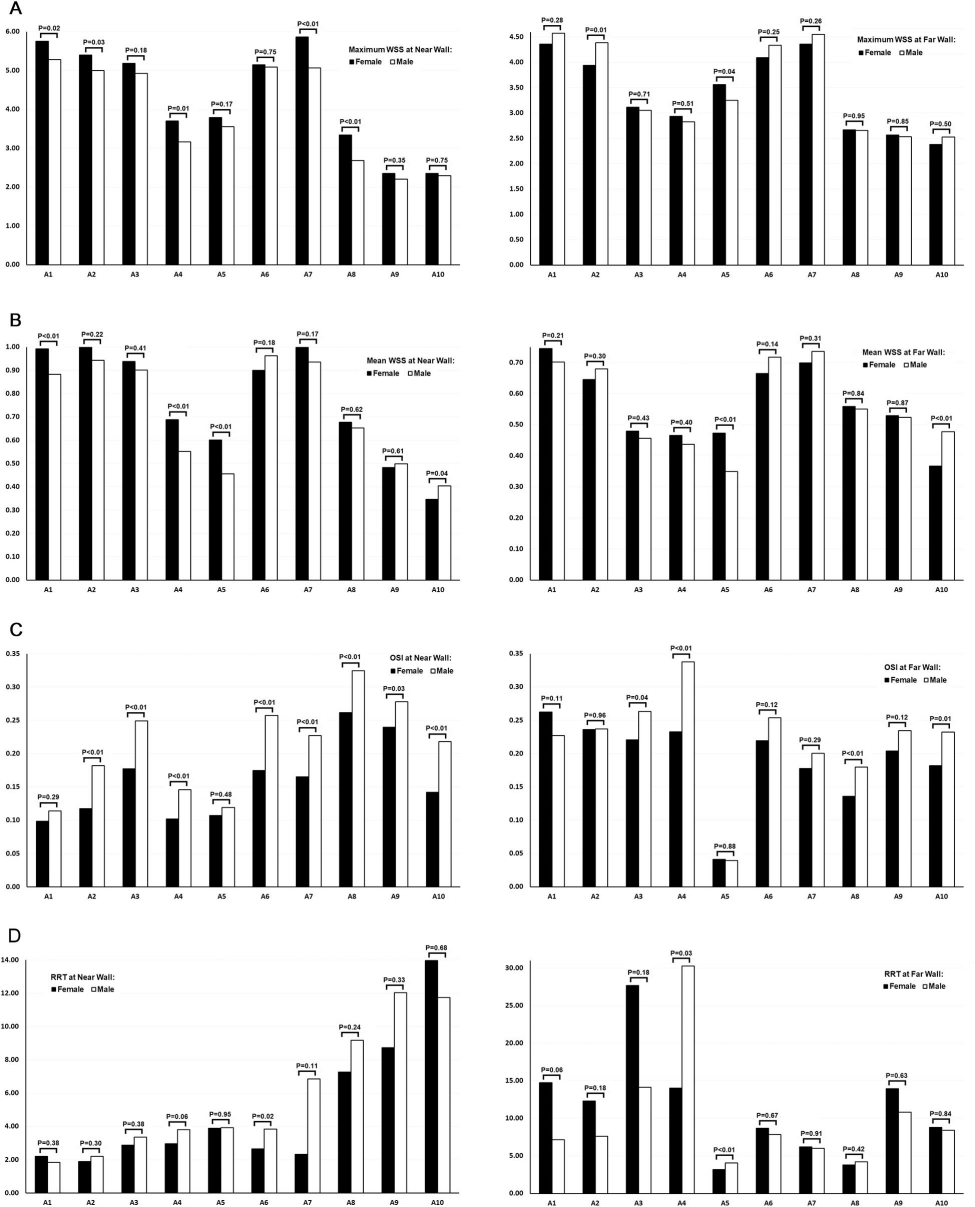
Comparison of WSS, OSI, and RRT values across the femoropopliteal artery in 222 lower limbs: females vs. males. **(A)** Maximum WSS at the near wall (left) and far wall (right); **(B)** Mean WSS at the near wall (left) and far wall (right); **(C)** OSI at the near wall (left) and far wall (right); **(D)** RRT at the near wall (left) and far wall (right). A1: Common femoral artery (CFA), 2 cm proximal to the femoral artery bifurcation; A2: CFA, one radius proximal to the bifurcation; A3: At the bifurcation; A4: Profunda femoris artery (PFA), one radius distal to the bifurcation; A5: PFA, 2 cm distal to the bifurcation; A6: Superficial femoral artery (SFA), one radius distal to the bifurcation; A7: SFA, 2 cm distal to the bifurcation; A8: SFA, 12.5 cm distal to the bifurcation; A9: SFA, 7.5 cm proximal to the popliteal skin crease; A10: Popliteal artery (PA), at the popliteal skin crease. WSS: wall shear stress, OSI: oscillatory shear index, RRT: relative residence time. Black bars represent data from female participants, and white bars represent data from male participants. *P*-values above the bars indicate the results of *t*-tests comparing female and male values at each location.

Values of maximum WSS, mean WSS, OSI, and RRT at the near and far walls of 10 locations along the femoropopliteal artery were compared between females and males (Table 3 and Figure 5).

**Table 3:** Comparison of WSS, OSI, and RRT values across the femoropopliteal artery in 222 lower limbs: females vs. Males.

Locations				CFA		Bifurcation	PFA		SFA				PA
				A1	A2	A3	A4	A5	A6	A7	A8	A9	A10
Maximum WSS (Pa)	Near wall	Female	Mean	5.75	5.40	5.19	3.70	3.79	5.15	5.86	3.34	2.35	2.35
			SD	1.58	1.37	1.50	1.82	1.42	1.41	1.42	1.64	1.13	1.30
		Male	Mean	5.28	5.00	4.93	3.16	3.55	5.09	5.07	2.68	2.20	2.29
			SD	1.42	1.34	1.32	1.43	1.10	1.49	1.30	1.27	1.11	1.60
		*P*-value		0.02	0.03	0.18	0.01	0.17	0.75	<0.01	<0.01	0.35	0.75
	Far wall	Female	Mean	4.36	3.94	3.12	2.94	3.56	4.10	4.36	2.67	2.57	2.38
			SD	1.42	1.36	1.33	1.21	1.27	1.33	1.22	1.05	1.32	1.35
		Male	Mean	4.57	4.39	3.05	2.83	3.25	4.34	4.55	2.66	2.53	2.53
			SD	1.40	1.20	1.27	1.18	1.00	1.65	1.28	1.27	1.35	1.71
		*P*-value		0.28	0.01	0.71	0.51	0.04	0.25	0.26	0.95	0.85	0.50
Mean WSS (Pa)	Near wall	Female	Mean	0.99	1.00	0.94	0.69	0.60	0.90	1.00	0.68	0.48	0.35
			SD	0.34	0.38	0.38	0.35	0.32	0.33	0.31	0.32	0.22	0.17
		Male	Mean	0.88	0.94	0.90	0.55	0.46	0.96	0.94	0.65	0.50	0.40
			SD	0.29	0.31	0.28	0.24	0.22	0.34	0.35	0.38	0.21	0.22
		*P*-value		<0.01	0.22	0.41	<0.01	<0.01	0.18	0.17	0.62	0.61	0.04
	Far wall	Female	Mean	0.75	0.65	0.48	0.47	0.47	0.66	0.70	0.56	0.53	0.37
			SD	0.29	0.27	0.24	0.25	0.27	0.25	0.26	0.32	0.27	0.15
		Male	Mean	0.70	0.68	0.46	0.44	0.35	0.72	0.74	0.55	0.52	0.48
			SD	0.23	0.22	0.20	0.24	0.19	0.27	0.28	0.30	0.26	0.20
		*P*-value		0.21	0.30	0.43	0.40	<0.01	0.14	0.31	0.84	0.87	<0.01
OSI	Near wall	Female	Mean	0.10	0.12	0.18	0.10	0.11	0.17	0.17	0.26	0.24	0.14
			SD	0.11	0.10	0.12	0.10	0.13	0.12	0.13	0.13	0.13	0.16
		Male	Mean	0.11	0.18	0.25	0.15	0.12	0.26	0.23	0.32	0.28	0.22
			SD	0.10	0.11	0.13	0.12	0.12	0.13	0.13	0.10	0.13	0.16
		*P*-value		0.29	<0.01	<0.01	<0.01	0.48	<0.01	<0.01	<0.01	0.03	<0.01
	Far wall	Female	Mean	0.26	0.24	0.22	0.23	0.04	0.22	0.18	0.14	0.20	0.18
			SD	0.15	0.15	0.16	0.16	0.08	0.16	0.16	0.12	0.14	0.15
		Male	Mean	0.23	0.24	0.26	0.34	0.04	0.25	0.20	0.18	0.23	0.23
			SD	0.17	0.15	0.15	0.15	0.08	0.16	0.16	0.13	0.14	0.15
		*P*-value		0.11	0.96	0.04	<0.01	0.88	0.12	0.29	<0.01	0.12	0.01
RRT	Near wall	Female	Mean	2.21	1.89	2.87	2.97	3.89	2.66	2.33	7.26	8.73	13.97
			SD	4.36	3.00	5.33	3.10	5.33	3.43	4.31	12.32	13.67	57.83
		Male	Mean	1.85	2.20	3.35	3.81	3.92	3.84	6.85	9.17	12.04	11.74
			SD	1.32	1.42	2.67	3.23	2.76	3.85	26.26	11.69	29.81	17.88
		*P*-value		0.38	0.30	0.38	0.06	0.95	0.02	0.11	0.24	0.33	0.68
	Far wall	Female	Mean	14.75	12.32	27.66	14.03	3.19	8.69	6.21	3.79	13.94	8.81
			SD	42.56	35.59	105.95	35.12	1.97	15.25	15.23	4.90	65.47	19.43
		Male	Mean	7.17	7.62	14.14	30.27	4.08	7.86	6.00	4.21	10.82	8.41
			SD	13.10	14.54	39.62	62.01	2.19	13.24	11.60	2.80	27.45	10.39
		*P*-value		0.06	0.18	0.18	0.03	<0.01	0.67	0.91	0.42	0.63	0.84

Data are presented as mean, standard deviation (SD). *P*-values were derived from *t*-test comparing values between females and males at the near and far walls of 10 locations. A1: Common femoral artery (CFA), 2 cm proximal to the femoral artery bifurcation; A2: CFA, one radius proximal to the bifurcation; A3: At the bifurcation; A4: Profunda femoris artery (PFA), one radius distal to the bifurcation; A5: PFA, 2 cm distal to the bifurcation; A6: Superficial femoral artery (SFA), one radius distal to the bifurcation; A7: SFA, 2 cm distal to the bifurcation; A8: SFA, 12.5 cm distal to the bifurcation; A9: SFA, 7.5 cm proximal to the popliteal skin crease; A10: Popliteal artery (PA), at the popliteal skin crease. WSS, wall shear stress; OSI, oscillatory shear index; RRT, relative residence time.

At the near wall, maximum WSS values were significantly lower in males than in females at 5 of the 10 locations. Mean WSS value at the near wall were significantly lower in males at 3 of the 10 locations, while at one location, values were significantly lower in females.

At the far wall, both maximum and mean WSS values were generally similar between sexes. For each parameter, one location showed a significantly lower value in males, and another showed a significantly lower value in females.

A greater number of locations with higher OSI values were observed in males—8 of 10 at the near wall and 4 of 10 at the far wall. No locations exhibited significantly higher OSI values in females at either wall.

RRT values, calculated from OSI and mean WSS measurements obtained via ultrasound, were significantly higher in males at the near wall of the proximal SFA (A6) and at the far wall of the profunda femoris artery (PFA, A4 and A5).

At the far wall of the femoral artery bifurcation, the mean RRT value was higher in females than in males (27.66 vs. 14.14); however, this difference was not statistically significant (*P* = 0.18). Interestingly, the median RRT value at the same location was lower in females than in males (4.73 vs. 5.98).

## Discussion

PAD is one of the most common clinical manifestations of atherosclerosis, capable of causing intermittent claudication, rest pain, and tissue loss, and may ultimately lead to amputation (32–34). The prevalence of atherosclerotic plaques and arterial occlusion varies throughout the lower limb arteries (35–38), likely due to differences in local anatomical and hemodynamic factors. In terms of hemodynamic forces, it is widely accepted that low and/or oscillatory WSS promotes atherosclerosis, whereas high WSS with low oscillation is protective (8).

In this study, we used ultrasound VFI to measure WSS and OSI. Compared to other imaging modalities such as MRI, CT, and intravascular elastography, ultrasound VFI is non-invasive, relatively inexpensive, and can be performed by ultrasound practitioners with minimal training.

Previous studies have demonstrated that WSS and OSI values vary between different arteries. In particular, WSS in the femoropopliteal artery is lower than in the carotid artery (28, 39, 40). This difference is attributed to the distinct functional roles of these vessels: muscular arteries, such as the femoropopliteal artery, primarily serve a conductive function, whereas elastic arteries, such as the carotid, store potential energy (39). As no validated thresholds are currently available to define high or low WSS, OSI, or RRT specifically for the femoropopliteal artery, this study used the average values of maximum WSS, mean WSS, OSI, and RRT across the femoropopliteal artery as reference values for comparison. It should be noted that these are not established clinical thresholds, and further studies are required to validate their use.

The results of the current study demonstrate that in the CFA, although both maximum and mean WSS values were relatively high at the anterior and posterior walls, significantly higher OSI and RRT values were observed at the posterior wall, while lower OSI and RRT values were recorded at the anterior wall. The more oscillatory nature of WSS at the posterior wall of the CFA may be associated with the higher prevalence of atherosclerotic plaques at this location, as reported in previous studies (35, 36, 38, 41).

The distal SFA at the adductor (Hunter) canal is the most common site for atherosclerotic plaques formation and arterial occlusion within the femoropopliteal artery (37, 42, 43). This may be attributed to the unique anatomic characteristics of the region, including: 1) the artery's limited ability to enlarge in response to mural thickening, due to the constraining fasciomuscular boundaries of the canal; 2) abrupt changes in mechanical stiffness as the artery passes through different tissue types—being tightly surrounded by muscles within the canal, then crossing the sharp edge of the aponeurosis of the adductor magnus to exit the canal and enter more compliant fatty tissue; and 3) repeated mechanical trauma during leg flexion (4, 37).

In addition to anatomical factors, local hemodynamic forces may also contribute to the formation of atherosclerotic lesions (36, 37). In this study, the distal SFA was the only segment of the femoropopliteal artery where significantly lower WSS (both maximum and mean), along with significantly higher OSI, were observed at both the near and far walls. Assuming that low WSS and high OSI promote atherosclerosis, this finding aligns with the high prevalence of atherosclerotic plaque formation and arterial occlusion in this region, as reported in previous studies. When atherosclerotic plaques develop from both the near and far walls and encroach into the arterial lumen, the likelihood of complete arterial occlusion may increase. Whether vessel mobility and/or flexibility influence hemodynamic forces in this region remains unclear.

RRT values, calculated based on OSI and mean WSS measured by the ultrasound system, exhibited a wide standard deviation and should be interpreted with caution. Because OSI and mean WSS values were rounded to two decimal places, OSI values between 0.495 and 0.500 were recorded as 0.50, and mean WSS values below 0.005 were recorded as 0.00. To prevent infinite values in the RRT calculation, OSI values of 0.50 were replaced with 0.4950, and mean WSS values of 0.00 were replaced with 0.0025. These adjustments may have led to overestimation of some RRT values and may partially explain the large standard deviation observed in the RRT data.

In comparing WSS, OSI, and RRT between females and males, our study revealed significantly lower maximum and mean WSS at multiple locations on the near wall of the femoropopliteal artery in males. Additionally, males exhibited significantly higher OSI at several locations on both the near and far walls, and higher RRT at the near wall of the proximal SFA and the far wall of the PFA, compared to females. These gender-related differences in hemodynamic forces within the femoropopliteal artery may contribute to the higher incidence of PAD observed in the male population (32). The mechanism underlying the differences in hemodynamics between sexes is not fully understood.

### Limitations

This study has several limitations. First, the participants were primarily staff members and their family members at Peking University International Hospital, which may not accurately represent the general population. Second, the study did not account for the configuration of the femoral artery bifurcation or other anatomical factors that may influence flow hemodynamics (44). Third, no ultrasound or clinical follow-up was performed; therefore, the clinical significance of the findings could not be evaluated. Lastly, the ultrasound system's rounding of WSS and OSI values to two decimal places may have introduced inaccuracies in the RRT calculation.

## Conclusion

Adults without PAD exhibit variations in WSS, OSI, and RRT along the femoropopliteal artery. Significantly lower and more oscillatory WSS were observed in the distal SFA at the adductor canal—a region consistently reported in the literature as having the highest prevalence of atherosclerotic lesions. Whether these hemodynamic patterns contribute to the development of atherosclerotic lesions later in life remains unclear and warrants further investigation through prospective studies.

## Data Availability

The raw data supporting the conclusions of this article will be made available by the authors, without undue reservation.
